# Cytogenetic characterization of *Hypostomus soniae* Hollanda-Carvalho & Weber, 2004 from the Teles Pires River, southern Amazon basin: evidence of an early stage of an XX/XY sex chromosome system

**DOI:** 10.3897/CompCytogen.v13i4.36205

**Published:** 2019-12-11

**Authors:** Luciene Castuera de Oliveira, Marcos Otávio Ribeiro, Gerlane de Medeiros Costa, Cláudio Henrique Zawadzki, Ana Camila Prizon-Nakajima, Luciana Andreia Borin-Carvalho, Isabel Cristina Martins-Santos, Ana Luiza de Brito Portela-Castro

**Affiliations:** 1 Faculdade de Ciências Biológicas e Agrárias, Universidade do Estado de Mato Grosso, Alta Floresta, Mato Grosso, Brazil; 2 Departamento de Biotecnologia, Genética e Biologia Celular, Universidade Estadual de Maringá, Maringá, Paraná, Brazil; 3 Departamento de Biologia, Acc., Universidade Estadual de Maringá, Maringá, Paraná, Brazil

**Keywords:** Fish cytotaxonomy, chromosome banding, rDNA FISH, chromosome polymorphism, Loricariidae

## Abstract

In the present study, we analyzed individuals of *Hypostomus soniae* (Loricariidae) collected from the Teles Pires River, southern Amazon basin, Brazil. *Hypostomus soniae* has a diploid chromosome number of 2n = 64 and a karyotype composed of 12 metacentric (m), 22 submetacentric (sm), 14 subtelocentric (st), and 16 acrocentric (a) chromosomes, with a structural difference between the chromosomes of the two sexes: the presence of a block of heterochromatin in sm pair No. 26, which appears to represent a putative initial stage of the differentiation of an XX/XY sex chromosome system. This chromosome, which had a heterochromatin block, and was designated proto-Y (pY), varied in the length of the long arm (q) in comparison with its homolog, resulting from the addition of constitutive heterochromatin. It is further distinguished by the presence of major ribosomal cistrons in a subterminal position of the long arm (q). The Nucleolus Organizer Region (NOR) had different phenotypes among the *H. soniae* individuals in terms of the number of Ag-NORs and 18S rDNA sites. The origin, distribution and maintenance of the chromosomal polymorphism found in *H. soniae* reinforced the hypothesis of the existence of a proto-Y chromosome, demonstrating the rise of an XX/XY sex chromosome system.

## Introduction

The Teles Pires River, in the southern Amazon basin, is the home of at least 36 species of Loricariidae, and five species of *Hypostomus* Lacépède, 1803 (Siluriformes, Loricariidae) (Ohara et al. 2017). *Hypostomus* is considered to be one of the taxonomically most complex genera of Neotropical fish due to its enormous diversity of morphology and body pigmentation patterns, with a total of 203 recognized species (Froese and Pauly 2019). The diversification of this genus appears to be closely related to changes in the chromosome complement, which include diploid numbers (2n) ranging from 52 in *H. emarginatus* (Artoni and Bertollo 2001) to 84 in *Hypostomus* sp. (Cereali et al. 2008). However, a phylogenetic analysis of mitochondrial DNA sequences (Montoya-Burgos 2003) indicated that *H. emarginatus* does not belong to the principal *Hypostomus* clade, which would mean that the lowest diploid number in the genus is 2n = 64, found in *H. cochliodon* (Bueno et al. 2013; Rubert et al. 2016), *H. faveolus* (Bueno et al. 2013), and *Hypostomus* sp. (Artoni et al. 1998; Fenerich et al. 2004; Milhomem et al. 2010).

A number of cytogenetic studies have examined various aspects of the differentiation of the *Hypostomus* karyotype, including complex karyotype evolution (Martinez et al. 2011; Alves et al. 2012; Pansonato-Alves et al. 2013; Bueno et al. 2014), heterochromatin polymorphism (Traldi et al. 2012; Baumgärtner et al. 2014), inter-individual chromosome polymorphism (Artoni and Bertollo 1999; Ferreira et al. 2019), and morphologically differentiated sex chromosomes (Artoni et al. 1998; Oliveira et al. 2015; Kamei et al. 2017). A range of sex chromosome systems found in 705 fish species are available in the Tree of Sex Consortium (2014) database. Differentiated sex chromosome systems are not very common in the loricariid catfishes, although simple (Alves et al. 2006; de Oliveira et al. 2007; Prizon et al. 2017) and multiple systems (Centofante et al. 2006; de Oliveira et al. 2008; Blanco et al. 2014) have been described in this family. In the genus *Hypostomus*, only a simple sexual chromosomal system has been described, with a XX/XY system being found in *H. ancistroides* and *H. macrops*, identified as *Plecostomus ancistroides* and *P. macrops*, respectively (Michele et al. 1977; Rocha-Reis et al. 2018), and a ZZ/ZW system in *Hypostomus* sp. G (Artoni et al. 1998), H. cf. plecostomus (Oliveira et al. 2015) and *H. ancistroides* (Kamei et al. 2017).

Highly differentiated sex chromosomes have been analyzed in a number of different groups of animals, although the initial stages of the evolution of sex chromosome systems have not often been described. Even so, an overview of the literature shows that our understanding of the various stages in the evolution of sex chromosome systems has increased progressively over time (Nanda et al. 1992; Bergero and Charlesworth 2009; Wright et al. 2016; Abbott et al. 2017; Kottler and Schartl 2018). The present study describes a karyotype with a putative initial stage of the differentiation of sex chromosomes in a population of *H. soniae* from the basin of the Teles Pires River, in southern Amazonia.

## Material and methods

We analyzed 17 *Hypostomus soniae* individuals (5 ♂ and 12 ♀) collected from urban streams located in Alta Floresta (9°54’30.82”S, 56°03’33.86”W; 9°53’50.47”S, 56°03’39.50”W; 9°53’30.53”S, 56°04’18.75”W), in Mato Grosso, Brazil. This area is part of the Teles Pires River drainage in the southern Amazon basin. The individuals were collected according to Brazilian environmental legislation (Collecting license MMA/IBAMA/SISBIO, number 31423-1). The individuals were anesthetized and euthanized by clove-oil overdose (Griffiths 2000). Voucher specimens were deposited in the ichthyological collection of the Núcleo de Pesquisa em Limnologia, Ictiologia e Aquicultura (Nupélia) of Universidade Estadual de Maringá (**UEM**) under catalogue number NUP 14991.

Chromosome preparations were obtained from kidney cells using the technique of Bertollo et al. (1978). The NORs were detected by impregnation with silver nitrate (AgNO_3_) (Howell and Black 1980). The constitutive heterochromatin was identified by C-banding (Sumner 1972), and stained with propidium iodide (Lui et al. 2012). Fluorescence *in situ* Hybridization (FISH) followed the protocol of Pinkel et al. (1986), using 18S rDNA probes from *Prochilodus argenteus* (Hatanaka and Galetti Jr. PM 2004), labeled with a Biotin Nick Translation kit, and 5S rDNA probes from *Leporinus elongatus* (Martins and Galetti Jr. PM 1999) labeled with a Digoxigenin Nick Translation kit. The chromosomes were classified according to Levan et al. (1964), i.e., metacentric (m), submetacentric (sm), subtelocentric (st), and acrocentric (a).

## Results

**Figure 1.**
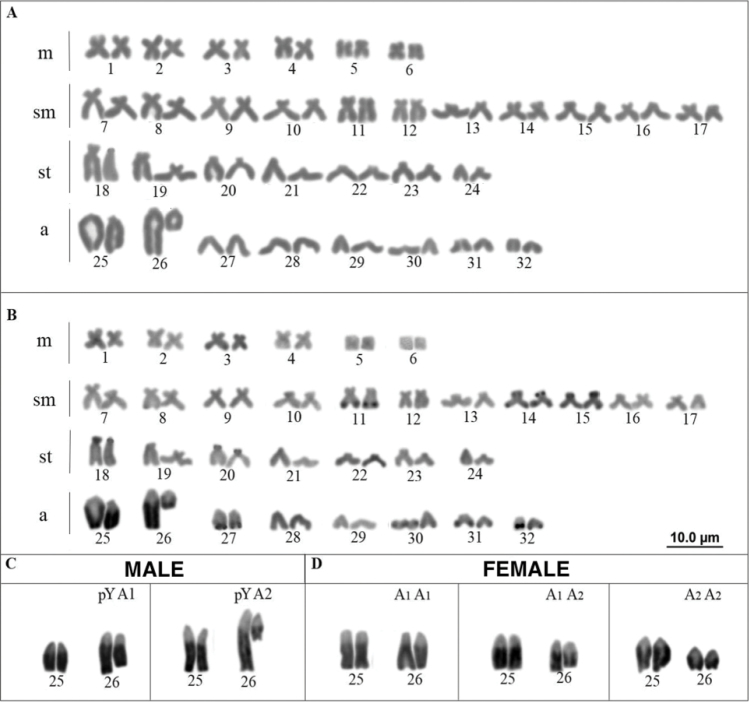
Karyotype of a male *Hypostomus soniae* obtained from **A** Giemsa-stained and **B** sequentially C-banded chromosomes. Variant chromosomes of pair No. 26, with pair No. 25 for comparison, in **C** males and **D** females. The dark regions in the chromosomes represent the heterochromatic blocks.

*Hypostomus soniae* has the diploid chromosome number of 2n = 64, fundamental number (FN) equal to 112, and a karyotype composed of 12m + 22sm + 14st + 16a chromosomes, in both males and females (Fig. 1A). Small heterochromatin blocks were observed in some chromosomes, primarily in the terminal regions, and conspicuous heterochromatic blocks were observed in the q arms of pairs Nos. 25 and 26 (Fig. 1B). The Giemsa staining and C-banding also revealed size heteromorphism between the homologs of pair No. 26 in the males and, to a lesser extent, in the females (Fig. 1A, B).

**Figure 2.**
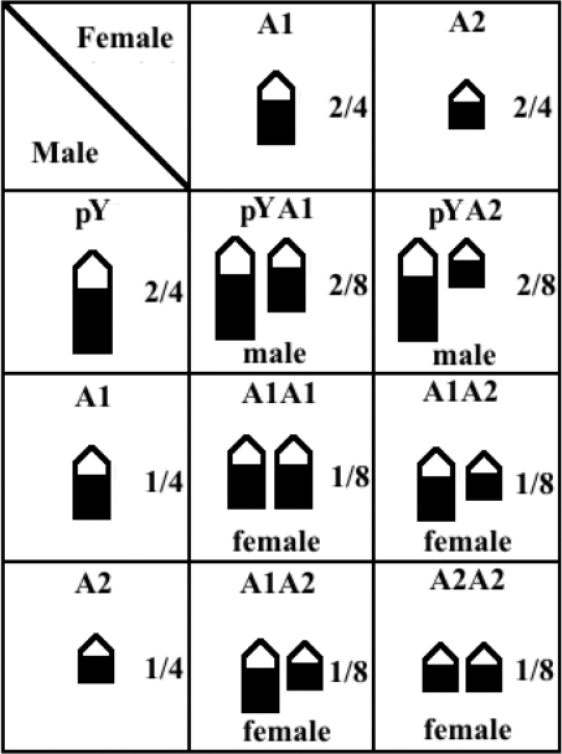
Combinations of the homologous pair No. 26 resulting from crossing males and females of the *Hypostomus soniae* study population. The dark regions in the chromosomes represent the heterochromatic blocks.

Pair No. 25 was highlighted for comparisons with pair No. 26, to determine more precisely the size difference between the homologs of the latter (Fig. 1C, D). This allowed us to identify three variant chromosomes that may correspond to pair No. 26 in the karyotypes of the individuals from the study population (Fig. 1C, D): (i) a chromosome larger than that of pair No. 25, which was found only in the males, and was designated pY (proto-Y); (ii) a chromosome similar in size to pair No. 25, designated A1, and (iii) a chromosome smaller than pair 25, designated A2. Considering a panmictic population, these chromosomes may form the following combinations for pair No. 26: in the males, pYA1 (found in 3 individuals) and pYA2 (2 individuals), whereas in the females, there are three possible combinations: A1A1 (3 individuals), A1A2 (6 individuals), and A2A2 (3 individuals) (Fig. 2).

**Figure 3.**
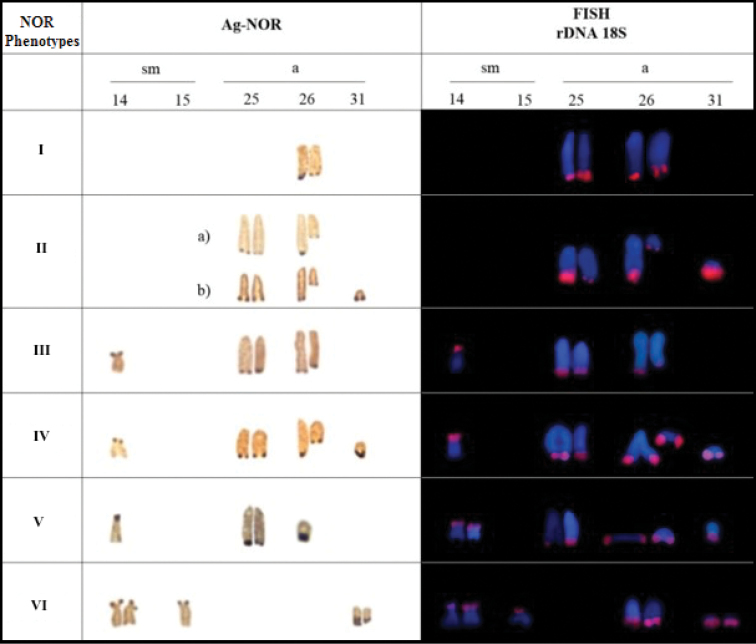
The Ag-NOR phenotypes observed in the karyotypes of *Hypostomus soniae*, detected by silver nitrate impregnation, FISH with 18S probes. The numbers 14, 15, 25, 26 and 31 represent the chromosomal pairs; sm = submetacentric; a = acrocentric.

The Ag-NOR-staining and FISH with the 18S rDNA probe revealed multiple nucleolus organizer regions (NORs) in a terminal portion of the short arms (p) of two pairs of sm chromosomes (Nos. 14 and 15) and in a terminal position of the q arms of three pairs of a chromosomes (Nos. 25, 26 and 31). Inter-individual variation in the 18S rDNA sites revelead six different phenotypes (Fig. 3). In all phenotypes, FISH revealed positive 18S rDNA sites in pair No. 26. The 18S rDNA sites corresponded to heterochromatin blocks in all cases.

## Discussion

*Hypostomus soniae* belongs to the “*H. cochliodon* species group” (Hollanda-Carvalho and Weber 2004) and has 2n = 64, similar to *H. cochliodon*, analyzed by Bueno et al. (2013) and Rubert et al. (2016), which is the lowest 2n found in the genus. Considering a basal 2n = 54 for the family Loricariidae (Artoni and Bertollo 2001), 2n = 64 would be the basal character for the genus *Hypostomus* (Bueno et al. 2014).

In *Hypostomus*, several cases of chromosomal polymorphism associated with the amplification of the heterochromatin, with or without ribosomal genes, have been reported (Artoni and Bertollo 1999; Traldi et al. 2012; Baumgärtner et al. 2014; Lorscheider et al. 2018), but in none of these cases was the polymorphism found in only one of the sexes. In the present paper, all the *H. soniae* individuals analyzed had the same karyotype structures, although differences were found between the sexes in pair No. 26, indicating a putative incipient process of sex chromosome differentiation. This differentiation pattern was supported by the presence of size heteromorphism in the heterochromatic block between the homologs of pair No. 26. This remarkable heterochromatin size polymorphism may indicate an early stage of the sex chromosome differentiation, where the chromosome with a large block of heterochromatin, designated here the proto-Y (pY), was observed only in the males. In the females, the corresponding homologs of pair No. 26 were also polymorphic, with one of the chromosomes having a heterochromatic block of medium size (designated A1) and the other (designated A2), a much smaller block. The detection of these variant chromosomes in both sexes reinforces the hypothesis of an initial process of heteromorphic sex chromosome formation, in which heterochromatinization plays a fundamental role.

The proto-Y chromosome in the genome of *H. soniae* is larger than the X chromosome, as observed in the Y chromosome of H. aff. ancistroides analyzed by Rocha-Reis et al. (2018). Thus, the larger size of the proto-Y chromosome may be the result of the apparent accumulation of heterochromatin, mediated by transposable elements, which may play an important role in the differentiation process, as observed in other species of fish (see Chalopin et al. 2015).

One other ancestral trait in the Loricariidae is the existence of a chromosome pair with NORs, which has been described in a number of fish species (Artoni and Bertollo 1996; Alves et al. 2005; Bueno et al. 2014; Rubert et al. 2016), including some species of the genus *Hypostomus* (Mendes-Neto et al. 2011; Rubert et al. 2011; Alves et al. 2012). Multiple NORs, as observed in *H. soniae* in the present study, are considered to be a derived characteristic, and are the most common pattern in the genus *Hypostomus* (Rubert et al. 2016; Brandão et al. 2018). In the “*H. cochliodon* group”, multiple NORs were noted in *H. cochliodon* from the Paraguay River basin (Rubert et al. 2016), although Bueno et al. (2014) observed a simple NOR in *H. cochliodon* individuals from the Paraná River basin. While *H. soniae* is part of the monophyletic “*H. cochliodon* species group”, the lack of data limits conclusions on which phenotype (simple or multiple NORs) is derived, because this feature has only been investigated in two species of this group, i.e., *H. soniae* (present paper) and *H. cochliodon* (Bueno et al. 2014; Rubert et al. 2016).

We observed inter-individual numerical variation in the Ag-NOR and 18S rDNA sites among the *H. soniae* individuals. This reflects the enormous mobility of the rDNA cistrons, and suggests the existence of dispersal mechanisms for these sites. The variation observed by silver staining is assumed to be the result of shifts in the control of the expression of ribosomal cistrons. The FISH 18S revealed that chromosome pair No. 26 was present in all of the different NOR phenotypes. These findings may reflect the transposition of rDNA genes, which had been located in pair No. 26, compared to the other chromosomes that bear major ribosomal cistrons. A similar hypothesis has been used to account for the variability in the number of NORs found in previous studies (Santi-Rampazzo et al. 2008; Porto et al. 2014). The presence of heterochromatin associated with all the ribosomal cistrons, as observed here, may indicate that mobile elements are part of the structure and organization of the adjacent heterochromatin found at these sites. While we did not investigate the presence of transposable elements (TEs) in the present study, these sequences are known to be associated with the 28S/18S rDNA in fish (Mandrioli et al. 2001; Symonová et al. 2013; Gouveia et al. 2017) and, more commonly, with the heterochromatin, including *Hypostomu*s (Pansonato-Alves et al. 2013).

The proto-sex chromosomes of *H. soniae* were also characterized by the presence of 18S rDNA cistrons. The association between the 18S rDNA sites and sex chromosomes has been reported in fishes (Artoni and Bertollo 2002, Chen et al. 2008; Cioffi and Bertollo 2010), including in the genus *Hypostomus* (Rocha-Reis et al. 2018). Repetitive sequences have been recorded at high frequencies in heterochromatic sex chromosomes and Chalopin et al. (2015) linked the evolution and emergence of sex chromosomes to the dynamics of the repeats and transposable elements. Therefore, the possible association of TEs with the ribosomal genes and adjacent heterochromatic blocks in pairs Nos. 25 and 26 in the *H. soniae* karyotype may indicate a possible link with TEs in the initial steps of the differentiation of the sex chromosomes.

## Conclusion

The data presented here on *H. soniae* include previously unpublished karyotypic arrangements, which represent an important contribution to future taxonomic studies of the *H. cochliodon* species group. In *Hypostomus*, the addition of heterochromatin to some chromosomes is the cause of polymorphisms resulting in different cytotypes, although this is the first cytological evidence of this mechanism emerging in sex chromosomes in this group. The apparent emergence of novel sex chromosomes in *H. soniae* makes this species an excellent potential model for the study of the differentiation and evolution of mechanisms of sexual determination, and the role of the accumulation and amplification of repetitive sequences in the origin and differentiation of sex chromosomes and its implications for the speciation process.
